# Adherence of Pharmaceutical Advertisements in Medical Journals to FDA Guidelines and Content for Safe Prescribing

**DOI:** 10.1371/journal.pone.0023336

**Published:** 2011-08-17

**Authors:** Deborah Korenstein, Salomeh Keyhani, Ali Mendelson, Joseph S. Ross

**Affiliations:** 1 Department of Medicine, Mount Sinai School of Medicine, New York, New York, United States of America; 2 Department of Health Evidence and Policy, Mount Sinai School of Medicine, New York, New York, United States of America; 3 HSR&D Research Enhancement Award Program and Geriatrics Research, Education, and Clinical Center, James J. Peters VA Medical Center, Bronx, New York, United States of America; 4 Weill Cornell Medical College, New York, New York, United States of America; 5 Section of General Internal Medicine, Department of Medicine, Yale University School of Medicine and Center for Outcomes Research and Evaluation, Yale-New Haven Hospital, New Haven, Connecticut, United States of America; Marienhospital Herne - University of Bochum, Germany

## Abstract

**Background:**

Physician-directed pharmaceutical advertising is regulated in the United States by the Food and Drug Administration (FDA); adherence to current FDA guidelines is unknown. Our objective was to determine adherence rates of physician-directed print advertisements in biomedical journals to FDA guidelines and describe content important for safe prescribing.

**Methods and Findings:**

Cross-sectional analysis of November 2008 pharmaceutical advertisements within top U.S.-based biomedical journals publishing original research. We excluded advertisements for devices, over the counter medications, and disease awareness. We utilized FDA guideline items identifying unique forms of advertisement bias to categorize advertisements as adherent to FDA guidelines, possibly non-adherent to at least 1 item, or non-adherent to at least 1 item. We also evaluated advertisement content important for safe prescribing, including benefit quantification, risk information and verifiable references. All advertisements were evaluated by 2 or more investigators, with differences resolved by discussion. Twelve journals met inclusion criteria. Nine contained pharmaceutical advertisements, including 192 advertisements for 82 unique products; median 2 per product (range 1–14). Six “teaser” advertisements presented only drug names, leaving 83 full unique advertisements. Fifteen advertisements (18.1%) adhered to all FDA guidelines, 41 (49.4%) were non-adherent with at least one form of FDA-described bias, and 27 (32.5%) were possibly non-adherent due to incomplete information. Content important for safe prescribing was often incomplete; 57.8% of advertisements did not quantify serious risks, 48.2% lacked verifiable references and 28.9% failed to present adequate efficacy quantification. Study limitations included its focus on advertisements from a single month, the subjectivity of FDA guidelines themselves, and the necessary subjectivity of determinations of adherence.

**Conclusions:**

Few physician-directed print pharmaceutical advertisements adhere to all FDA guidelines; over half fail to quantify serious risks. The FDA could better protect public health by creating new more objective advertisement guidelines requiring transparent presentation of basic safety and efficacy information.

## Introduction

Advertising is a crucial component of pharmaceutical industry marketing around the world and advertising in biomedical journals is estimated by industry to be its most profitable marketing strategy [1]. Furthermore, physician exposure to advertisements has been associated with increased prescribing of advertised drugs [2]. Beyond marketing, proponents of physician-directed advertisements, mostly from within the pharmaceutical industry, emphasize the important role advertisements play in educating physicians and other prescribers about new drugs [3]. However, critics have raised concerns about the quality of the information presented in these physician-directed advertisements, including a focus on relative, not absolute, benefit [3]–[5] and poor referencing [6]–[8]. Although systematic assessments of advertisement content to promote evidence-based prescribing have been limited [9], [10], one previous study found that physician-directed advertisements did not promote guideline-adherent patient care [11], and critics have argued that advertising may harm patients and adversely impact public health [12].

In the United States, the Food and Drug Administration (FDA) is charged with regulating all pharmaceutical marketing through its Division of Drug Marketing and Advertising (DDMAC). However, ensuring advertisement adherence is challenging and FDA resources dedicated to this task are limited. DDMAC's fiscal year 2008 budget of $9 million [13] is dwarfed by the pharmaceutical industry's $58 billion marketing budget [14]. While the FDA can require that companies change or remove non-adherent advertisements, given DDMAC's size and the volume of marketing materials it regulates, it would be unreasonable to expect each individual pharmaceutical advertisement to be reviewed.

Acknowledging this regulatory challenge, the FDA has recently asked physicians to report non-adherent or misleading advertisements through its “Bad Ad” program, explaining that “With your valuable assistance, FDA can be more effective in limiting the number of misleading promotional messages directed to health care professionals” [15]. This program may prove useful in identifying grossly misleading advertisements; however there has been no systematic assessment of the adherence of physician-directed advertisements to FDA regulations in the last 20 years [16] and none to current standards, which were last updated in 2001.

Therefore, our objectives were to determine adherence to the 2001 FDA guidelines among current print advertisements directed at physicians within biomedical journals and to describe content important for safe prescribing. We performed a cross-sectional analysis of advertisements for prescription pharmaceuticals in top biomedical journals published in issues over a single month and hypothesized that rates of non-adherence to FDA guidelines would be high and that many advertisements would not present complete information important for safe prescribing.

## Methods

### Study Sample

We conducted a cross-sectional analysis of advertisements for prescription pharmaceuticals in top U.S. biomedical journals. We included all U.S.-based journals with a Thomson Institute for Scientific Information impact factor >10 that published original research. We excluded journals publishing only review articles and journals without a print edition.

We identified all advertisements for prescription pharmaceuticals published in any issue during November 2008. We excluded advertisements for devices and over-the counter medications and those designed to heighten awareness of particular diseases without mention of disease pharmacotherapy. Multiple different advertisements for the same product were all included but duplicate advertisements for the same prescription pharmaceutical were included only once in our sample.

### Advertisement Assessment

Our advertisement assessment tool contained 36 items, of which 3 described basic characteristics of the advertised drug (e.g. is the drug a combination pill?), 4 described basic features of the advertisement (e.g. are there efficacy claims?), 21 were adapted from FDA guidelines [16], described below, and 8 related to advertisement content in support of safe prescribing, described below (see data collection tool in Figure S1).

### Advertisement Assessment: FDA Guidelines

Our advertisement assessment tool included items based on FDA Prescription Drug Advertising Guidelines. ^16^FDA guidelines describe 21 unique items used to classify advertisements as “false, lacking in fair balance, or otherwise misleading” [16]. We classified the items covered by these FDA guidelines into four non-exclusive content domains: drug safety (4 items), drug efficacy (10 items), references (7 items), and other items, which included quotes (3 items), statistics (2 items), headlines (1 item), and photographs (1 item).

### Advertisement Assessment: Safe Prescribing and Other Content

Our assessment tool also included descriptive items and items related to content important for safe prescribing. Safe prescribing content items were based on prior literature [5], [6], [10], [15] and accepted standards for the clinical application of data [17]. These items focused on the presence of verifiable references and the quantification of risks and benefits with inclusion of appropriate efficacy numbers, defined as information adequate to calculate number needed to treat [17], or survival times in the case of chemotherapeutic agents. To further clarify the presentation of risks and benefits, we recorded the presence of a black box warning for each advertised drug and whether the drug places patients at risk for death or serious morbidity. In addition, we categorized advertised drugs into four product types based on indications for their use in the treatment of conditions related to cardiovascular/diabetes, psychiatric, hematology/oncology or other diseases.

### Advertisement Review and Evaluation

Prior to initiating our reviews, we examined advertisements which had received FDA warning letters in 2008 (of which none was in our sample) to determine the approach of FDA to the regulations. All advertisements were reviewed between January and December of 2009. Each advertisement was reviewed by at least 2 of 3 investigators (DK, SK and JSR) and we attempted to determine adherence in a way that was slightly more conservative than the approach of the FDA itself (i.e. less likely to rate advertisements as non-adherent). During advertisement review, we referred directly to the FDA language embedded within our assessment tool (see data collection tool in the Figure S1) and utilized objective measures when possible. We discussed each item until consensus was reached and were intentionally conservative when rating the presence of FDA-described features, erring toward classifying advertisements as guideline adherent. Table S1 describes our approach to FDA item scoring.

Options for abstraction item responses included “yes” and “no”, with additional options of “not applicable” and “not sure” where appropriate. We coded the majority of items based only on the content of the advertisement as presented in the journal, but for items related to drug status as a first-line agent we used the 2008 Tarascon Pocket Pharmacopoeia® (Jones and Bartlett; Sudbury, MA) and for items related to the completeness of references and drug-associated risk of death or serious morbidity we searched Medline using the internet site pubmed.gov, considering only data published before October 2008. To establish black box warnings, we utilized the internet site *blackboxrx.com*, considering only warnings issued prior to October 2008.

Advertisements were categorized as adherent, non-adherent, or possibly non-adherent to FDA guidelines. Advertisements were considered *adherent* if they contained none of the 21 features used by FDA to classify advertisements as misleading, *non-adherent* if they contained one or more of the features used by FDA to classify advertisements as misleading, and *possibly non-adherent* if there were no features clearly defining a misleading ad but at least 1 of those items for which information was incomplete.

### Statistical Analysis

We used descriptive statistics to report rates of advertisement adherence, non-adherence, and possible non-adherence, overall and categorized by item content (i.e., safety, efficacy, references, other) and by pharmaceutical product indication (i.e., cardiovascular/diabetes, psychiatric, hematology/oncology or other). We then used Chi-square tests to test for differences in these advertisement non-adherence rates. Two reviewers (DK and JSR) independently reviewed a randomly selected 5% sub-sample of advertisements in order to measure inter-rater reliability and agreement beyond chance (kappa). Analyses were performed using JMP 7.1 (SAS Institute, Inc., Cary, NC). All statistical tests were 2-tailed and used a type I error rate of 0.05.

## Results

### Advertisement Sample

Twelve biomedical journals met our inclusion criteria. Three contained no advertisements for prescription pharmaceuticals (CA, Journal of the National Cancer Institute and Gastroenterology); 9 contained advertisements that were included in our study: Annals of Internal Medicine (n = 14 advertisements), Archives of General Psychiatry (n = 5), Blood (n = 36), Circulation (n = 9), Hepatology (n = 2), JAMA (n = 10), Journal of the American College of Cardiology (n = 25), Journal of Clinical Oncology (n = 56), and the New England Journal of Medicine (n = 35). We reviewed a total of 193 advertisements published during November 2008, of which one was a correction of a previous advertisement and was excluded from further analysis. Our final sample included 89 unique advertisements for 82 products. Seven products were represented in 2 unique advertisements each; the remaining 103 non-unique advertisements were duplicates, with a median of 2 advertisement appearances per product (range 1–14). Thirty-six manufacturers advertised products in our sample, with a median of 3 advertisements (range 1–21) and 2 unique advertisements (range 1–7) per company. Of the 89 unique advertisements, 6 (6.7%) were “teaser” advertisements, presenting only the drug name without additional content; the remaining 83 (93.3%) were full advertisements and were the focus of our analysis (Table 1).

**Table 1:** Sample Description and Characteristics of Physician-Directed Print Advertisements Published in High-Impact Biomedical Journals during November 2008.

.	N (%)
Total advertisements reviewed	193
Unique advertisement	89 (46.1)
Duplicate advertisement	104 (53.9)
Unique “Teaser” advertisement^a^^,^ b	6 (6.7)
Unique Full Advertisement^a^	83 (93)
**Characteristic of advertised drugs among Unique Full Advertisements** c	
Combination pill	6 (7.2)
Second line agent	18 (21.7)
**Product indication type** c	
Hematology/Oncology	43 (51.8)
Cardiovascular/Diabetes	17 (20.5)
Psychiatric	7 (9.4)
Other	16 (19.3)

a Among 89 unique advertisements.

b Providing only product name.

c Among 83 full unique advertisements.

Six of the 83 (7.2%) full unique advertisements were for “combination–pill” pharmaceutical products and 18 (21.7%) were for second line agents. More than half of advertised products (n = 43) had an indication for use for hematology/oncology-related treatment, 17 for cardiovascular/diabetes-related treatment, 7 for psychiatric-related treatment and 16 for other indications (Table 1). Our inter-rater reliability with regard to ratings of FDA guideline items was excellent, with 90.3% agreement and a kappa of 0.860.

### Adherence to FDA guidelines

Among the 83 unique full advertisements, 15 (18.1%) were fully adherent to FDA guidelines, 41 (49.4%) were non-adherent to at least 1 FDA mandated item, and 27 (32.5%) were possibly non-adherent due to incomplete information (Table 2). Among the 41 advertisements that were non-adherent to at least 1 FDA item, the mean number of items to which the advertisement was non-adherent was 1.37 (SD = 0.73, range 1–4) and among the 68 advertisements that were non-adherent or possibly non-adherent, the mean number of items to which the advertisement was non-adherent or possibly non-adherent was 3.51 (SD = 2.62, range 1–11). Forty (48.2%) advertisements were categorized as possibly non-adherent due to an absence of verifiable references in support of efficacy or safety claims. The most common FDA criteria to which advertisements were non-adherent or possibly non-adherent are shown in Table 3, with descriptions of specific non-adherent advertisements.

**Table 2:** Rates of adherence to FDA guidelines among physician-directed print advertisements in high-impact biomedical journals, overall by FDA guideline item content.^a^

	Adherent, No. (%)	Non-adherent,^b^ No. (%)	Poss. non-adherent,^c^ No. (%)
**Overall**	15 (18.1)	41 (49.4)	27 (32.5)
**FDA Guideline Item Content**			
Safety	58 (69.9)	9 (10.8)	16 (19.3)
Efficacy	39 (47.0)	18 (21.7)	26 (31.3)
References	47 (56.6)	16 (19.3)	20 (24.1)
Other Issues	53 (63.9)	23 (27.7)	7 (8.4)
Quotes (n = 2)	1 (50.0)	1 (50.0)	0 (0)
Statistics^d^ (n = 24)	22 (91.7)	0 (0)	2 (8.3)
Headlines (n = 80)	67 (83.8)	8 (10.0)	5 (6.3)
Photographs (n = 41)	33 (80.5)	8 (19.5)	0

Notes: NA = Non-adherence; RR = Relative Risk; CI = Confidence Interval.

a Content domains were non-exclusively categorized.

b Defined as non-adherent to at least 1 item in the category.

c Defined as no non-adherent items in the category, but a response of “not sure” for at least 1 item in the category.

d Includes statistical testing and pooling of data questions.

**Table 3:** Top 3 FDA guideline items to which advertisements were non-adherent and top 3 FDA guideline items to which advertisements were possibly non-adherent^a^.

FDA Guideline Item	Non-Adherent No.	Possibly Non-Adherent No. (%)	Combined No. (%)	Example
**Most common ** ***non-adherent*** ** items**				
“Contains literature references or quotations that are significantly more favorable to the drug than has been demonstrated by substantial evidence”	10 (12.0)	10 (12.0)	20 (24.1)	An advertisement for a drug for leukemia did not mention data related to standard of care drugs which are available generically.
“Uses headline, subheadline, or pictoral or other graphic matter in a way that is misleading”	8 (9.6)	5 (6.0)	13 (15.7)	An advertisement for a drug for advanced lung cancer depicts a healthy-looking man apparently windsurfing on the open sea
“Represents or suggests that drug dosages properly recommended for … certain classes of patients or disease(s) are safe and effective for … other classes of patients or diseases”	8 (9.6)	0 (0)	8 (9.6)	An advertisement for a hypertension medication which is not recommended in guidelines as a first or second line agent claims to be first-line for patients likely to need more than 1 agent
**Most common ** ***possibly non-adherent*** ** items**				
“Uses literature, quotations, or references that purport to support (a) …claim but…do not support the claim or have relevance”	4 (4.8)	22 (26.5)	26 (31.3)	An advertisement for a drug for hematologic malignancy contains efficacy claims citing data on file. The cited study is now published and had an inappropriate comparison group, so it does not truly support the efficacy claim.
“Presents information from a study in a way that implies that the study represents larger or more general experience with the drug than it actually does”	1 (1.2)	20 (24.1)	21 (25.3)	Multiple advertisements were “possibly non-adherent” to this item because there were no verifiable references.
“Contains references…that misrepresent the effectiveness of a drug by failure to disclose…information…concerning concomitant therapy…(or) placebo effect”	5 (6.0)	17 (20.5)	22 (26.5)	An advertisement for a medication for pulmonary hypertension has claims for which the supporting data was an open-label trial; thus results may be related to placebo effect.

a Other items had identical rates, so the item with the highest combined rate is displayed.

There were statistically significant differences in non-adherence rates to FDA guidelines by item content. Nine (10.8%) advertisements were non-adherent to at least 1 FDA item focused on safety, whereas 18 (21.7%) advertisements were non-adherent to at least 1 FDA item focused on efficacy (p = 0.06)16 (19.3%) to at least 1 FDA item focused on references (p = 0.13), and 23 (27.7%) to at least 1 FDA item focused on other issues (p = 0.006) (Table 3). Among items focused on other issues, rates of non-adherence varied (Table 2).

There were statistically significant differences in non-adherence rates to FDA guidelines by product indication category. Four of 16 (25.0%) advertisements for “other use” pharmaceutical products were non-adherent to at least 1 FDA item, whereas 26 of 43 (60.5%) advertisements for hematology/oncology products were non-adherent to at least 1 FDA item (p = 0.01), 9 of 17 (52.9%) advertisements for cardiovascular/diabetes products were non-adherent to at least 1 FDA item (p = 0.10), and 2 of 7 (28.6%) advertisements for psychiatric products were non-adherent to at least 1 FDA item (p = 0.99).

### Safe Prescribing Content

Many advertised drugs in our sample presented potential risks to patients, with 31 (37.3%) containing black box warnings and 33 (39.8%) placing patients at risk for death or serious morbidity. However, when evaluating advertisement content important for safe prescribing, we found that many physician-directed advertisements lacked important information. The majority (n = 48; 57.8%) did not quantify serious risks and 24 (28.9%) failed to present appropriate numbers to quantify benefits. Thirty-six advertisements (43.4%) referenced “data on file” and 40 (48.2%) lacked verifiable references, due either to an absence of any references or to the presence of references only to “data on file” or the product's package insert (Table 4).

**Table 4:** Content related to safe prescribing among physician-directed print advertisements in high-impact biomedical journals.

Quality issue	No. (%)
Does not quantify serious risks	48 (57.8)
Does not quantify benefits	23 (27.7)
No appropriate numbers are presented (regarding efficacy claims)^a^	24 (28.9)
Black box warning present	31 (37.3)
Claims are out of context^b^	19 (22.9)
No verifiable references presented	40 (48.2)

a Absolute risk reduction or NNT, or survival times for oncology advertisements.

b Comparing to placebo rather than active control, or focusing on surrogate outcomes.

## Discussion

In this study we found that nearly half of physician-directed advertisements fail to adhere to at least one FDA guideline regulating content. Physician-directed advertisements contained bias with regard to a wide variety of issues across content areas addressed by FDA guidance; there was no single problem that was consistently identified for non-adherence. In addition, we found that advertisements do a poor job of conveying basic information necessary for safe prescribing, with the majority failing to quantify serious risks, more than one quarter failing to quantify benefits and nearly half providing no verifiable references. Our study is the first in nearly 20 years to provide a systematic assessment of the adherence of U.S. advertisements to FDA guidance and provides context to inform the FDA's new “bad ad” program.

Despite the high rates of FDA non-adherence, the mean number of biased features in each advertisement was low and most advertisements we reviewed satisfied the majority of FDA-guidelines despite a general absence of content important for safe prescribing. FDA guidelines, though detailed, do not target many of the ad features most important for providing prescribers with useful information. For instance, FDA emphasizes avoiding frankly false information and balancing efficacy and safety information [19] but does little to encourage the presentation of useful and accurate information. An advertisement containing no specific efficacy claim, no quantification of drug safety and no verifiable references would adhere fully to FDA guidelines, despite presenting no practical information for clinicians. We found many such advertisements in our sample; for example an advertisement for a new chemotherapeutic agent presented the indication, the claim that it was the first in a new drug class, a vague image, and safety information with no quantification of effect and no references.

The expectation that advertisements should inform rational prescribing by presenting complete drug safety and efficacy information may be unrealistic since advertisements primarily serve a marketing function [20] and are not designed to train physicians to properly prescribe. However, proponents of physician-directed advertising emphasize the important role advertisements play in educating physicians and other prescribers about new drugs [3], and it is clear that advertisements do serve to influence prescribing by providing information to physicians. While the majority of physicians deny that advertisements inform their prescribing [21], marketing research has consistently shown that journal advertising is the most profitable form of drug marketing, with an estimated return on investment of $5 for every dollar spent [1]. Furthermore, in some cases exposure to physician-directed advertising has been shown to be associated with less effective, lower quality prescribing decisions [22]. It is clear that prescribers are ultimately responsible for the safety and efficacy of drugs they prescribe but few doctors recognize the extent to which they are influenced [23]. Physicians should ensure that their prescribing is informed by the clinical literature and not by marketing materials. However, given the high risk associated with many advertised drugs, any tendency of advertisements to encourage inappropriate prescribing may pose dangers to patients. If FDA is truly committed to improving prescribing and protecting the health of the public it should demand that content important for safe and effective prescribing be consistently presented in physician-directed advertisements.

A major challenge of our study was performing advertisement evaluations given the subjectivity of the current FDA guidelines. For instance, the FDA describes that advertisements are misleading if they use “a quote or paraphrase out of context to convey a false or misleading idea” or “headline, subheadline, or pictoral or other graphic matter in a way that is misleading”, without providing explicit definitions. We created objective criteria for many items but scoring still required subjective determinations. Our conservative approach to subjective items may have underestimated the true non-adherence rate of the advertisements we evaluated, which may explain why the rate of advertisement non-adherence in our study is lower than the 92% non-adherence rate demonstrated in a 1990 study of journal advertisements directed at physicians [15].

There are several other important limitations to our study. First, we sampled advertisements from a single month in 2008, so we cannot comment on trends in advertisement characteristics over time. We focused on high-impact biomedical journals publishing original research, excluding lower-impact and more narrowly focused publications and those publishing clinical reviews, which may also be frequently read among clinicians. We may have missed important differences in advertisements published in these other types of publications and we expect the volume of advertisements to have been greater in these journals. However, we suspect that major differences in advertising content are unlikely since pharmaceutical company advertisements are generally created for use in many journals, not just the highest-impact journals. We did not evaluate advertisements presented in other contexts (e.g. direct mailings to physicians) or other media (e.g. online). Data on the influence of newer marketing strategies is limited but journal advertisements appear to remain influential [24]. In addition, while visual aspects of advertisements may be highly influential, a full content analysis of advertisement imagery was beyond the scope of this paper. We did note a few patient photos which were misleading enough to lead to FDA non-adherence, but it is likely that other advertisements utilized more subtle imagery to imply more efficacy or broader patient applicability; thus our analysis may underestimate the misleading nature of some advertisements. Further, the items we measured to evaluate information important for safe prescribing were not validated, although the importance of quantifying benefits and risks is generally accepted [18] and the concepts evaluating safe prescribing are straightforward.

Our findings have important policy implications. The FDA has demonstrated a desire to improve the quality of pharmaceutical advertisements, most recently by enlisting doctors to review advertisements through its “Bad Ad” program. FDA should now move to update and simplify its guidelines for physician advertisements to facilitate the review process. Updated FDA guidelines should be straightforward and objective, and should include requirements that physician advertisements present clear risk quantification, absolute benefit information, description of the appropriate population to receive the drug, and verifiable references, either to the peer-reviewed published literature or to studies registered within federally-sponsored internet sites such as *ClinicalTrials.gov* in the case of unpublished data. These new guidelines might be applicable only to physician-directed advertisements, with separate guidance for DTC material, with greater focus on safety issues. Enforcement would still be necessary, as in other nations advertisements have failed to adhere to new straightforward requirements [25]. However, new objective requirements would serve to enhance prescribing and public health by improving the quality, accuracy and transparency of advertisements, simplify at least part of the FDA review process, and facilitate physician participation in reporting “bad” advertisements.

In conclusion, we found low rates of adherence to FDA guidelines among physician directed pharmaceutical advertisements and inadequate information for safe prescribing. Advertising is a form of free speech and is a constitutionally protected right of industry [26]. However, in keeping with its mission to “protect the public health by helping to ensure that prescription drug information is truthful, balanced, and accurately communicated” [27] the FDA can ensure that the information provided in advertisements leads to safe and effective prescribing on the part of clinicians. Current FDA guidelines are subjective and challenging to enforce and do not emphasize transparency and the inclusion of basic information relevant to prescribing. Increased enforcement of the current regulations would help improve advertisements, but the subjectivity of the guidelines presents a substantial barrier. We are hopeful that an update in FDA regulations, with increased emphasis on the transparent presentation of basic safety and efficacy information, might improve the quality of information provided in physician-directed pharmaceutical advertisements.

## Supporting Information

Table S1 FDA Guideline Item Content Domains and Approach to Determining Advertisement Adherence.
(DOC)

Figure S1 Master guide for advertisement review.
(DOC)
