# Supplementary material for: Adherence of Pharmaceutical Advertisements in Medical Journals to FDA Guidelines and Content for Safe Prescribing
Source: PLoS One. 2011 Aug 17;6(8):e23336. doi: 10.1371/journal.pone.0023336 (PMC3157354; doi:10.1371/journal.pone.0023336)
Supplement: Table S1 — FDA Guideline Item Content Domains and Approach to Determining Advertisement Adherence. (DOC) [file pone.0023336.s001.doc]

**Table S1:** FDA Guideline Item Content Domains and Approach to Determining Advertisement Adherence.

| **FDA Guideline Item Content Domain** | **Number of items** | **Example of FDA language describing non-adherence** | **Approach to Adherence^a^** |
| --- | --- | --- | --- |
| Efficacy | 4 | “Contains a drug comparison that represents…that a drug is… more effective than another drug…when (that) has not been demonstrated by substantial evidence.” | Defined “substantial evidence” as a blinded randomized controlled trial or systematic review published before October 2008 |
| Safety | 10 | “Fails to present information relating to side effects and contraindications with a prominence and readability reasonably comparable with the presentation of information regarding effectiveness” | Defined reasonably comparable “readability” and “prominence” if information was legible and on the main page(s) of the advertisement |
| References | 7 | “Uses literature, quotations, or references that purport to support (a)…claim but…do not support the claim or have relevance.” | References in support of claim defined as published studies in agreement with the content of the claim |
| Quotes | 3 | “Uses a quote or paraphrase out of context to convey a false or misleading idea” | Quotes defined as “false or misleading” if the meaning in the ad differed substantially from the original intent because of selective presentation of information |
| Statistical Testing and Data Pooling | 2 | “Uses ‘statistics’…in a way that suggests…that such ‘statistics’ are valid if they are not.” | Defined “statistics” as a p-value, confidence interval, or the presence of the word “statistical” in the ad text |
| Headlines and Pictures | 2 | “Uses headline, subheadline, or pictoral or other graphic matter in a way that is misleading” | Defined “misleading” patient photos as those that portrayed patients that clearly would not be drug candidates, e.g. a patient with advanced cancer windsurfing |
| Tables and Graphs | 2 | “Uses tables and graphs to distort or misrepresent the relationships, trends, differences or changes among the variables” | Defined misleading graphs as those with interrupted, distorted or unlabeled axes or with bars misrepresenting the magnitude of effect |

^a^ Definitions applied to all FDA guideline items.
