# Supplementary material for: Adherence of Pharmaceutical Advertisements in Medical Journals to FDA Guidelines and Content for Safe Prescribing
Source: PLoS One. 2011 Aug 17;6(8):e23336. doi: 10.1371/journal.pone.0023336 (PMC3157354; doi:10.1371/journal.pone.0023336)
Supplement: Figure S1 — Master guide for advertisement review. (DOC) [file pone.0023336.s002.doc]

**Figure S1. Master guide for advertisement review**

*NB- light-gray shaded boxes contain FDA criteria for ads that “may be” false, lacking in fair balance, or otherwise misleading. Open boxes refer to issues that per the FDA reflect ads that “are false…” Items without “document language” are descriptive and are not mentioned by the FDA*

| **Document Language** | **Checklist item** | **#** | **Answer** |
| --- | --- | --- | --- |
|  | Does the ad state that the drug is a second line agent? | 1 | 1=Yes  2=No |
|  | Is the ad for a combination pill? | 2 | 1=Yes  2=No |
| “Offers a combination of drugs for …patients (with) a condition amenable to treatment by any of the components RATHER than limiting the indications to (a situation in which) the fixed combination is indicated | For combination pills, would ONE of the components suffice for the presented indication? | 3 | 1=Yes  2=No  3=Not sure  9=NA |
|  | **Are side effects described in a “second page” of the ad?** | 4 | 1=Yes  2=No |
|  | Does the Rx put patients at risk of permanent disability or mortality? | 5 | 1=Yes  2=No  9=NA |
|  | Does the Rx carry a black box warning? | 6 | 1=Yes  2=No  9=NA |
|  | **Are safety issues addressed in the text of the ad?** | 7 | 1=Yes  2=No |
| **(e.g., 1 in 10 people have Adverse Event)** | Does the ad quantify serious risks in the text of the ad? | 8 | 1=Yes  2=No  9=NA |
| “Contains a drug comparison that represents…that a drug is **SAFER** or more effective than another drug …when (that) has not been demonstrated by substantial evidence” | Are there unsupportable **safety** claims related to comparisons with other drugs? | 9 | 1=Yes  2=No  3=Not sure  9=NA |
|  | Are common side effects described in the text of the ad? | 10 | 1=Yes  2=No  9=NA |
| “Fails to present information relating to side effects and contraindications with a prominence and readability reasonably comparable with the presentation of information relating to effectiveness | Are common side effects and contraindications presented clearly and legibly? | 11 | 1=Yes  2=No  3=Not sure  9=NA |
|  | **Are there any efficacy claims in the ad?** | 12 | 1=Yes  2=No |
| **(e.g., 20% decrease in mortality)** | Does the ad quantify benefits? | 13 | 1=Yes  2=No  9=NA |
|  | Were any efficacy data or numbers presented? | 14 | 1=Yes  2=No  9=NA |
| **(Absolute Risk Reduction or NNT)** | Were appropriate efficacy data or numbers presented? | 15 | 1=Yes  2=No  3=Not sure  9=NA |
| “Contains a drug comparison that represents…that a drug is safer or **MORE EFFECTIVE** than another drug …when (that) has not been demonstrated by substantial evidence” | Are there unsupportable **efficacy** claims related to comparisons with other drugs? | 16 | 1=Yes  2=No  3=Not sure  9=NA |
|  | Were **efficacy** claims made out of context?  a) comparing to placebo rather than active-control  b) focusing on surrogate rather than clinical outcomes | 17 | 1=Yes  2=No  9=NA |
| “Contains …data or conclusions from non-clinical studies…which suggests they have clinical significance when in fact no such …significance has been demonstrated | Are **efficacy** claims based on non-clinical data? | 18 | 1=Yes  2=No  3=Not sure  9=NA |
|  | Were any **efficacy** claims made that were not supported by rigorous evidence (e.g., a blinded RCT)? | 19 | 1=Yes  2=No  3=Not sure  9=NA |
| “Uses literature…(to) suggest…conditions of drug use that are not approved or permitted | Are there unapproved **efficacy** claims? | 20 | 1=Yes  2=No  3=Not sure  9=NA |
| “Uses statements or representations that a drug differs from…a named drug or category or drugs…in a way that suggests falsely or misleadingly or without substantial evidence or …clinical experience that the advertised drug is safer or more effective than such other drug | Does the ad make unfounded claims regarding competitor drugs or does it claim inaccurately that advertised drug differs from other drugs?  [specific comparison] | 21 | 1=Yes  2=No  3=Not sure  9=NA |
|  | **Are there references?** | 22 | 1=Yes  2=No |
|  | Does the ad reference data on file? | 23 | 1=Yes  2=No  9=NA |
|  | Does the ad *exclusively* reference data on file? | 24 | 1=Yes  2=No  9=NA |
| “Contains literature references or quotations that are significantly more favorable to the drug than has been demonstrated by substantial evidence or substantial clinical experience | Do references misrepresent the entirety of data in support of drug efficacy?  (e.g., selectively references only “favorable” studies) | 25 | 1=Yes  2=No  3=Not sure  9=NA |
| “Contains a representation or suggestion that a drug is safer than…has been demonstrated…, by selective presentation of information. | Do references misrepresent the entirety of data in support of drug safety?  (e.g., selectively references only “safe” studies) | 26 | 1=Yes  2=No  3=Not sure  9=NA |
| “Uses a statement by a recognized authority that is …favorable…but fails to refer to concurrent or more recent unfavorable data or statements from the same authority on the same subject | Are citations from authorities out of date or incomplete?  (e.g., individual of authority – surgeon general – or an organization – American Heart Association) | 27 | 1=Yes  2=No  3=Not sure  9=NA |
| “Uses a study of normal individuals without disclosing that the subjects were normal” | Were cited studies performed on non-diseased people without disclosing that fact?  (e.g., study of normal people) | 28 | 1=Yes  2=No  3=Not sure  9=NA |
| “Presents information from a study in a way that implies that the study represents larger or more general experience with the drug then it actually does” | Are the claims more general than the referenced studies would support?  (e.g., study of sub-population) | 29 | 1=Yes  2=No  3=Not sure  9=NA |
| “Contains references…that misrepresent the effectiveness of a drug by failure to disclose…information…concerning concomitant therapy, …(or) placebo effect. | Do claims related to referenced studies fail to explain that effect may have been related to placebo effect or other factors?  (e.g., study has no control group) | 30 | 1=Yes  2=No  3=Not sure  9=NA |
| “Uses literature, quotations, or references that purport to support (a)…claim but …do not support the claim or have relevance | Do references actually support the claim?  (e.g., reference is unrelated study or not verifiable trial data) | 31 | 1=Yes  2=No  3=Not sure  9=NA |
| “Uses data favorable to a drug derived from patients treated with dosages different from those recommended in approved…labeling” | Does the ad reference unapproved dosages?  (e.g., references studies of higher or lower dosages than is generally used) | 32 | 1=Yes  2=No  3=Not sure  9=NA |
|  | **Are there quotes?** | 33 | 1=Yes  2=No |
| “Uses a quote or paraphrase out of context to convey a false or misleading idea” | Are quotes misrepresented by having been taken out of context? | 34 | 1=Yes  2=No  3=Not sure  9=NA |
|  | **Was statistical testing discussed in the text?** | 35 | 1=Yes  2=No |
| “Uses erroneously a statistical finding of ‘no significant difference’ to claim clinical equivalence or to …conceal…potential…real clinical difference” | Is a claim of “no significant difference” used erroneously?  (e.g., does the text state “as good as” when statistical equivalence testing was not used) | 36 | 1=Yes  2=No  3=Not sure  9=NA |
|  | **Was there pooling of data?** | 37 | 1=Yes  2=No |
| “Uses ‘statistics’ …derived from pooling data from various insignificant or dissimilar studies in a way that suggests…that such ‘statistics’ are valid if they are not or that they are derived from large or significant studies… | Is there inappropriate or misleading pooling of data? | 38 | 1=Yes  2=No  3=Not sure  9=NA |
|  | **Are there headlines or sub-headlines in the ad?** | 39 | 1=Yes  2=No |
| “Uses headline, subheadline, or pictoral or other graphic matter in a way that is misleading? | Are the headlines or sub-headlines misleading? | 40 | 1=Yes  2=No  3=Not sure  9=NA |
|  | **Are there pictures used in the ad?(photos)** | 41 | 1=Yes  2=No |
| “Represents or suggests that drug dosages properly recommended for…certain classes of patients or disease(s) are safe and effective for…other classes of patients or diseases. | Does the picture used in the ad misrepresent the appropriate population to receive the drug? | 42 | 1=Yes  2=No  3=Not sure  9=NA |
|  | **Is there EFFICACY data presented in text, tables, or graphs?** | 43 | 1=Yes  2=No |
| “Uses tables or graphs to distort or misrepresent the relationships, trends, differences or changes among the  variables | Do the tables or graphs DISTORT or MISREPRESENT the data? | 44 | 1=Yes  2=No  3=Not sure  9=NA |
